# The Translated Amino Acid Sequence of an Insertion in the Hepatitis E Virus Strain 47832c Genome, But Not the RNA Sequence, Is Essential for Efficient Cell Culture Replication

**DOI:** 10.3390/v13050762

**Published:** 2021-04-26

**Authors:** Johannes Scholz, Alexander Falkenhagen, Reimar Johne

**Affiliations:** German Federal Institute for Risk Assessment, Max-Dohrn-Straße 8-10, 10589 Berlin, Germany; Johannes.Scholz@bfr.bund.de (J.S.); alexander.falkenhagen@bfr.bund.de (A.F.)

**Keywords:** hepatitis E virus, cell culture, reverse genetics system, genome insertion, hypervariable region, G1634R mutation

## Abstract

The hepatitis E virus (HEV) can cause hepatitis E in humans. Recently, the occurrence of HEV strains carrying insertions in their hypervariable genome region has been described in chronically infected patients. The insertions originate from human genes or from the HEV genome itself. Although their distinct functions are largely unknown, an involvement in efficient cell culture replication was shown for some strains. The HEV strain 47832c, originally isolated from a chronically infected transplant patient, carries a bipartite insertion composed of HEV genome duplications. Here, several mutants with deletions and substitutions of the insertion were generated and tested in cell culture. Complete deletion of the insertion abolished virus replication and even a single glycine to arginine substitution led to reduced cell culture growth. A mutant encoding a frameshift of the inserted sequence was not infectious, whereas a mutant carrying synonymous codons in this region replicated similar like the wild type. Substitution of the insertion with the S17 insertion from HEV strain Kernow C1-p6 did not result in viable virus, which might indicate strain- or cell type-specificity of the insertions. Generally, the translated amino acid sequence of the insertion, but not the RNA sequence, seems to be responsible for the observed effect.

## 1. Introduction

Infections with the hepatitis E virus (HEV) can cause acute and chronic hepatitis in humans with a mortality rate generally ranging from 0.2 to 4%, but which can reach up to 13%, as reported in a recent study [1,2]. In developing counties, large epidemic outbreaks have been described, which are linked to waterborne HEV infections. In contrast, mainly sporadic hepatitis E cases occur in industrialized countries, which are caused by zoonotic virus transmission from animals to humans [3,4]. The clinical symptoms range from asymptomatic infections to severe disease courses. While fever, vomiting, diarrhea and icterus are common symptoms of an acute hepatitis E, extrahepatic manifestations including neurologic disorders have also been attributed to HEV infection [5]. Chronic HEV infections with rapidly progressing liver disease have been described in immunosuppressed patients [5]. In many countries in Europe, the notified hepatitis E cases steeply increased during the last years [6].

The four main human-pathogenic HEV genotypes are classified into the family *Hepeviridae* and the species *Orthohepevirus A* [7]. Genotypes (GTs) 1 and 2 only infect humans and are transmitted through the fecal-oral route, mainly via contaminated drinking water. In contrast, GT3 and GT4 are zoonotic and are mainly transmitted via contact with pigs and wild boars or consumption of meat products derived from these animals [8].

HEV appears as a non-enveloped particle in the feces and as a quasi- enveloped particle in serum and cell culture supernatant [9]. The genome consists of a 5′-capped and 3′-polyadenylated positive-oriented single-stranded RNA, which contains three open reading frames (ORFs) [10,11]. The capsid protein is encoded by ORF2 and a multifunctional phosphoprotein by ORF3 [12,13]. ORF 1 codes for a non-structural polyprotein with domains of a methyltransferase, a papain-like cysteinprotease, an RNA helicase and an RNA-dependent RNA polymerase (RdRp) [14]. Additional regions of the ORF1 include the domains X and Y as well as the hypervariable region (HVR), the functions of which are largely unknown. The HVR codes for a proline-rich region of highly variable amino acid sequence when compared between different HEV strains [15]. It has been suggested to be involved in host range adaptation [16] and efficiency of RNA replication [17]. In addition, several insertions have been described within the HVR of strains derived from chronically infected patients [18]. These insertions originate from human host genes or from the HEV genome and can vary in length resulting in longer HVRs that range between 228 and 315 nt [19]. Although the functions of the insertions are not known thus far, a strong effect on the efficiency of replication in cell culture has been shown for some specific HEV strains [20,21]. For example, the insertion of the GT3 strain HEV Kernow C1-p6 is derived from the human ribosomal S17 gene and has been shown to be essential for efficient growth of this strain in HepG2/C3A cells as well as to enhance cell culture replication of the GT1 strain SAR-55 when inserted into its HVR [22].

The HEV strain 47832c is a GT3 strain originally isolated in Germany from a chronically infected kidney transplant patient [23]. This strain efficiently replicates in A549/D3 cells and has been used widely in basic and applied studies [24,25,26,27,28,29]. It carries a bipartite insertion in its HVR, which originates from duplications of an adjacent part of its HVR and of a part of its RdRp region. Interestingly, the latter part is derived from an RdRp region, for which the mutation G1634R (G/R) has been identified. This mutation often appears in patients after prolonged ribavirin therapy and conferred faster cell culture replication when introduced into the HEV strain Kernow C1-p6 [30,31]. In strain 47832c, this region is therefore present twice, in both cases having a glycine at the respective position. Thus far, the distinct function of the HVR insertion in strain 47832c is not known, but an involvement in cell culture growth has been proposed [23].

In the presented work, the significance of the insertion in the HVR of strain 47832c for its replication in cell culture was determined and functionally important parts of the insertion were analyzed. Using a recently established reverse genetics system for this virus strain [32], several mutants carrying deletions, sequence substitutions or point mutations in the respective region were generated and tested for their growth in cell culture. By this, it is shown that the presence of the original translated amino acid sequence of the insertion, but not its nucleotide sequence, is crucial for efficient replication of the strain in cell culture. Even the introduction of the single G/R mutation into the insertion dramatically decreases the replication efficiency. Substitution of the insertion with the S17 sequence of strain Kernow C1-p6 did not result in rescue of infectious virus in cell culture. The results indicate that the effect of the insertions may be strain- or cell type-specific and that the insertion of strain 47832c operates at the protein level.

## 2. Materials and Methods

### 2.1. Cells and Viruses

The cell line A549/D3 is a subclone of the human lung adenocarcinoma cell line A549, which shows a higher susceptibility to infection with the HEV strain 47832c [33]. These cells were used for all infection experiments. The cell line BSR T7/5 was kindly provided by Karsten Tischer (Free University of Berlin, Germany). This cell line is a derivative of baby hamster kidney (BHK) cells, which stably expresses a T7 RNA polymerase [34]. It was used for transfection with genomic plasmids carrying a T7 RNA polymerase promotor sequence.

The HEV strain 47832c is a GT3c strain, which was originally isolated from a chronically infected kidney transplant patient from Germany [23]. The cDNA clone p47832mc (Genbank accession number MN756606) contains the complete genome of strain 47832c under control of a T7 RNA polymerase promotor [32] and can be used for the generation of replicating virus by transfection into BSR T7/5 cells.

### 2.2. Generation of Plasmids Carrying Deletions and Sequence Substitutions in the 47832c Genome

**Figure 1:**
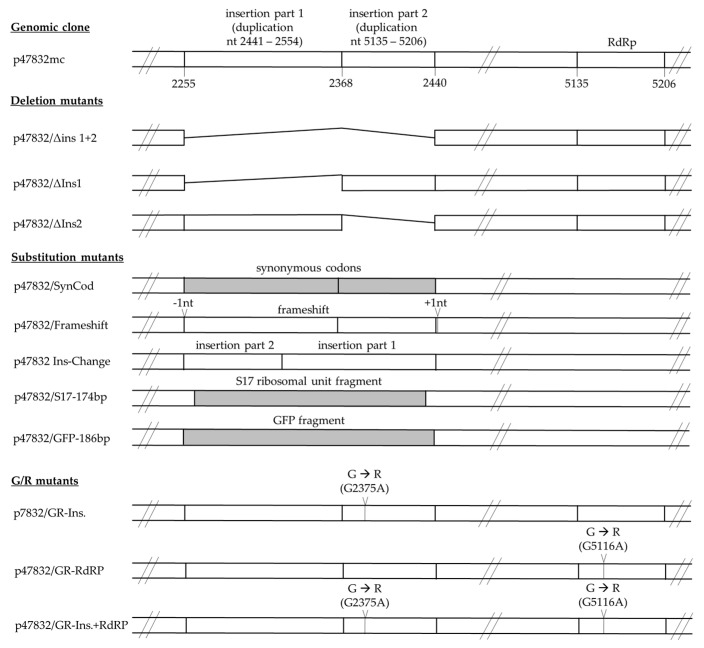
Schematic presentation of the generated constructs containing deletions or substitutions in the insertion of HEV strain 47832c. The original insertion is composed of two adjacent duplications from different regions of the HEV genome. The indicated numbers refer to the nucleotide positions of the genome of strain 47832c (GenBank acc.-no. KC618403.1). RdRp—RNA-dependent RNA polymerase, GFP—green fluorescent protein, S17 human ribosomal protein S17. Details of the constructs are described in the Text and in the Supplementary Table S1.

The mutants were generated by modification of plasmid p47832mc using methods as previously described [32]. Briefly, DNA fragments containing the desired deletions, sequence substitutions or point mutations (schematic view in Figure 1 and details in Supplementary Table S1) were synthesized by commercial providers (Integrated DNA Technologies, Coralville, IA, USA or GenScript Biotech B.V., Leiden, The Netherlands). Longer synthesized fragments were cloned into the vector pCR4-TOPO (Thermo Fisher Scientific, Waltham, MA, USA). Either the cloned fragments or the synthesized fragments directly were digested with specific restriction endonucleases (Thermo Fisher Scientific, Waltham, MA, USA or New England Biolabs (NEB), Schwalbach, Germany) and the resulting fragments were substituted with the respective fragments of the digested plasmid p47832mc. The sequences of the resulting genomic plasmids were validated by NGS, as described previously [32]. In addition, the substituted regions of the plasmids were validated using Sanger sequencing (Eurofins Genomics Germany GmbH, Ebersberg, Germany). The sequencing primers are provided in Supplementary Table S2. The sequences of the plasmids have been deposited at the GenBank database with accession numbers MW573944-MW573955.

### 2.3. Generation of Infectious Viruses from Plasmids

The generation of infectious viruses from the plasmids was performed as described [32]. Plasmids were purified using the Plasmid Midi Kit (Qiagen, Hilden, Germany). BSR T7/5 cells were seeded in 6-well plates at 24 h before transfection in DMEM supplemented with 10% fetal calf serum (FCS), 1× non-essential amino acid solution (NEAA), 1% l-glutamine (200 mM) and 1% gentamicin (10 mg/mL) (all cell culture reagents provided by Pan-Biotech GmbH, Aidenbach, Germany) in order to reach over 90% confluence. A total of 8 µg of the respective plasmid was mixed together with the two vaccinia virus capping plasmids pCAG D1R (1 µg) and pCAG D12L (1 µg) (Addgene plasmids #89160 and #89161), 250 µL OptiMEM (Thermo Fisher Scientific, Waltham, MA, USA) and 30 µL TransIT^®^-LT1 transfection reagent (MIRUS Bio, Madison, WI, USA). After incubation for 25 min, the mixture was added directly to the supernatant of BSR T7/5 cells and incubated for 24 h at 37 °C and 5% CO_2_. After 24 h, the medium was exchanged with DMEM supplemented with 2% FCS, 1× NEAA, 1% l-glutamine (200 mM) and 1% gentamicin (10 mg/mL), and the cells were incubated for additional six days at 34.5 °C and 5% CO_2._ Thereafter, cells were frozen/thawed three times, centrifuged at 5000× *g* for 10 min and the resulting supernatant was used to infect fresh A549/D3 cells. These A549/D3 cells had been seeded in 6-well plates two weeks prior to infection and had been incubated at 37 °C with 5% CO_2_ in MEM Eagle medium supplemented with 10% FCS, 1× NEAA, 1% l-glutamine (200 mM) and 1% gentamicin (10 mg/mL), with one complete medium change after 1 week. Infection was done with 1 mL supernatant prepared as described above for 1 h at room temperature. The supernatant was then removed and fresh medium was added. After 2 weeks of incubation at 37 °C and 5% CO_2_ in DMEM medium supplemented with 10% FCS, 1× NEAA, 1% l-glutamine (200 mM) and 1% gentamicin (10 mg/mL) and complete medium changes at each 3rd or 4th day, the culture supernatant was obtained (without freezing and thawing) and 1 mL was used for a second passage of fresh A549/D3 cells for 2 weeks, which was performed similarly to the 1st passage. The cells from the first and second virus passage were analyzed by immunofluorescence as described below. Aliquots of the supernatant were taken at day 0 (after removal of infection supernatant and adding of fresh medium), day 7 (before medium exchange) and day 14 (at the end of virus passage). The supernatants were stored at −20 °C and analyzed by RT-qPCR as described below.

### 2.4. Immunofluorescence Assay

The immunofluorescence assay was performed as described previously [24]. Briefly, cells were fixed with acetone/methanol (1:1) for 30 min at 4 °C. After removal of the fixation solution, cells were washed once with PBS and blocked with PBS containing 1% FCS for 1 h at 37 °C. The blocking solution was discarded and an HEV capsid protein-specific rabbit hyperimmune serum (kindly provided from Rainer Ulrich, Friedrich-Loeffler-Institute, Greifswald-Insel Riems, Germany; 1:500 dilution in PBS containing 1% FCS) was added. After 1 h incubation at 37 °C, the antibody was removed and cells were washed three times with PBS. FITC-conjugated anti-rabbit IgG (Sigma, Deisenhofen, Germany; 1:1000 in PBS containing 1% FCS) was added and the cells were incubated for 1 h at 37 °C. The secondary antibody was discarded and the cells were washed twice with PBS and once with distilled water. The cells were mounted with Roti^®^-Mount Fluor Care DAPI (Carl Roth, Karlsruhe, Germany) and analyzed using an Axio Observer Z1 microscope (Carl Zeiss, Oberkochen, Germany).

### 2.5. Reverse Transcription—Quantitative PCR (RT-qPCR)

RNA was isolated from the supernatant aliquots using the NucliSens^®^ EasyMag^®^ system (Biomérieux, Nürtingen, Germany). Twenty microliters of the RNA preparation was subjected to DNase I digest (Roche, Basel, Switzerland) for 2 h at 37 °C, followed by an inactivation step for 5 min at 75 °C. A quantitative real-time reverse transcription polymerase chain reaction (RT-qPCR) was performed in a 7500 real-time cycler (Applied Biosystems, Foster City, CA, USA) using an established HEV-specific primer/probe system [35] together with an in-house RNA standard [36] to determine the RNA copy numbers in the analyzed samples. The standard curve and the RNA copy numbers were calculated using the 7500 Fast Software v. 2.3 (Applied Biosystems, Foster City, CA, USA).

### 2.6. Generation of Persistently Infected Cell Lines

A549/D3 cells were infected with supernatants of transfected BSR T7/5 cells, which were generated as described above (see Section 2.3). In order to generate persistently infected cell lines, fresh A549/D3 cells were seeded in T25 cell culture flasks two weeks prior to infection and were incubated at 37 °C with 5% CO_2_ in MEM Eagle medium supplemented 10% FCS, 1% NEAA, 1% l-glutamine (200 mM) and 1% gentamicin (10 mg/mL), with a complete medium change after one week. After infection with 1 mL supernatant for 1 h at room temperature, fresh DMEM medium supplemented with 10% FCS, 1% NEAA, 1% l-glutamine and 1% gentamicin was added and the cells were incubated at 37 °C with 5% CO_2_. After one week, the medium was completely changed. After an additional week, the medium was removed, the cells were split at a ratio of 1:2 using trypsin (0.25%)/EDTA (0.1%) and seeded into new T25 cell culture flasks. Cells were incubated at 37 °C and 5% CO_2_ in the same medium as above for one week before the splitting procedure was repeated. After 4 cell passages by splitting, an aliquot of cells was seeded into a 6-well plate, which was analyzed after one week by immunofluorescence. In order to validate that the generated viruses contained the modified HVRs or RdRp gene, viral RNA was extracted from cell culture supernatants as described above and the genome regions containing the HVR or RdRp gene were amplified using the primers specified in Supplementary Table S2 and the Qiagen One-step RT-PCR kit (Qiagen, Hilden, Germany). Amplicons were extracted after agarose gel electrophoresis using the Monarch Gel Extraction Kit (NEB, Schwalbach, Germany). Sanger sequencing was performed by a commercial provider (Eurofins Genomics Germany GmbH, Ebersberg, Germany) using the PCR primers.

### 2.7. Viral Growth Kinetics

The replication kinetics of the generated viruses were compared with those of the wildtype virus, which was generated using the plasmid p47832mc. To determine the infectious units of the viruses, an endpoint dilution assay was performed as described [24]. Briefly, 1:5 dilution series of viral supernatants from passage one were set up in DMEM without supplements. Fresh A549/D3 cells grown in 96-well plates were used for infection, which was done similarly as described above. Infectious titers were analyzed at two weeks after infection by performing an immunofluorescence assay as described above. Thereafter, the viruses were diluted to titers of 7 × 10^1^ focus-forming units/mL using fresh DMEM minimal medium. The growth kinetics assays were performed with the titer-adjusted viruses by infection of A549/D3 cells in 6-well plates as described above, for a total time-period of 21 days. Every 3rd or 4th day, 500 µL aliquots were retrieved from the culture supernatants and stored. At the same time, the medium was completely changed. The aliquots were analyzed by RT-qPCR as described above.

### 2.8. Statististical Analyses

Analysis of significant differences between growth behaviors of viruses derived from the p47832 mutants compared to the wildtype virus p47832mc was performed by an unpaired, heteroscedastic Student’s *t*-test using Microsoft Excel Software. Values were considered as significant when the *p*-value was below 0.05 (*p* < 0.05).

## 3. Results

### 3.1. Generation of Constructs Containing Deletions or Substitutions in the Insertion of p47832mc

In order to investigate the function of the insertion within the HVR of strain 47832c, several constructs were generated (Figure 1). The genomic clone p47832mc, which contains the 47832c wild-type virus genome, was used as positive control. Three deletion mutants, in which either the whole insertion (p47832/Δins1+2) or only part 1 (p47832/Δins1) or only part 2 (p47832/Δins2) of the insertion were deleted, were generated. In mutant p47832/SynCod, the codons of the insertion were replaced by synonymous codons, thus changing the nucleotide sequence, but not the translated amino acid sequence of the insertion. In addition, mutant p47832/Frameshift retained the nucleotide sequence while the translated amino acid sequence of the insertion was changed. This was realized by a deletion of the first nucleotide of the insertion, which led to a frameshift of the coding sequence (without generation of any stop codon), and an insertion of one nucleotide at the end of the insertion, which restored the original reading frame for the remaining part of ORF1. Mutant p47832/Ins-Change contained both parts of the insertion in changed order. In mutant p47832/GFP-186bp, the insertion sequence was substituted by a GFP fragment of the same length, whereas mutant p47832/S17-174bp contained the inserted S17 sequence from HEV strain Kernow C1-p6 instead of the original insertion. In addition, mutants were generated which contained point mutations leading to a single glycine-to-arginine substitution in the context of the originally described G1634R mutation. As this region is present two times in strain 47832c, one mutant containing a single substitution in the insertion (p47832/GR-Ins), one mutant containing the substitution only in the RdRp region (p47832/GR-RdRp), and one mutant containing both substitutions (p47832/GR-Ins+RdRp) were generated. A more detailed description of the mutants is given in the Supplementary Table S1. The sequences have been deposited at the GenBank database with accession numbers MW573944-MW573955. The sequences of all generated plasmids were verified by Sanger sequencing and NGS, showing that the expected sequences were present in all cases.

### 3.2. Effect of Deletions within the Insertion Region of the HVR

**Figure 2:**
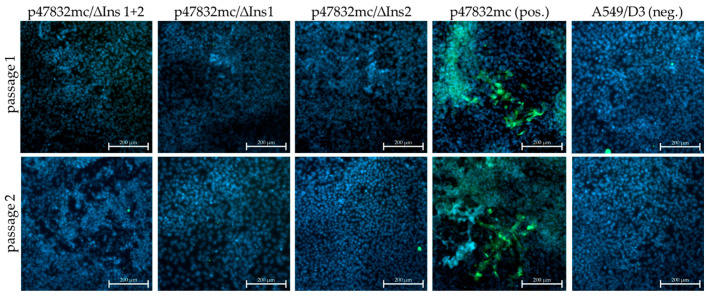
Testing of constructs containing deletions in the insertion region of the HVR of strain 47832c. The indicated constructs were transfected into BSR T7/5 cells and the resulting cell lysate was used for passaging on A549/D3 cells. The A549/D3 cells from the first (passage 1) and second (passage 2) passage are shown, which were analyzed by immunofluorescence using an HEV capsid protein-specific antiserum. Green signal: HEV capsid protein, blue signal: nuclear DAPI staining. Scale bar: 200 µm.

The constructs p47832/Δins1+2, p47832/Δins1 and p47832/Δins2 (Figure 1) were tested for their ability to generate infectious virus after transfection of cell cultures. The applied procedure was identical to that previously established for the genomic plasmid p47832mc [29], which was also used here as a positive control. Briefly, the purified plasmids were transfected together with two helper plasmids encoding capping enzymes into BSR T7/5 cells, in which capped genomic RNAs are synthetized that can serve as templates for protein expression and for generation of infectious virus. Thereafter, lysates of the transfected cells were used for infection of A549/D3 cells, which are highly susceptible to infection with strain 47832c. After a first passage, the resulting culture supernatant was used for the infection of fresh A549/D3 cells representing the second virus passage. Infected cells were thereafter detected by immunofluorescence using an antiserum directed against the HEV capsid protein. Whereas HEV-infected cells were readily detected in the passages of virus generated from the control plasmid p47832mc, no infected cells could be identified in either passage using the constructs with deletions in the insertion region (Figure 2). This indicates that the insertion sequence is essential for the generation of an efficiently replicating virus in the applied system. These results were confirmed twice.

### 3.3. Effect of Substitutions of the Insertion Region of the HVR

**Figure 3:**
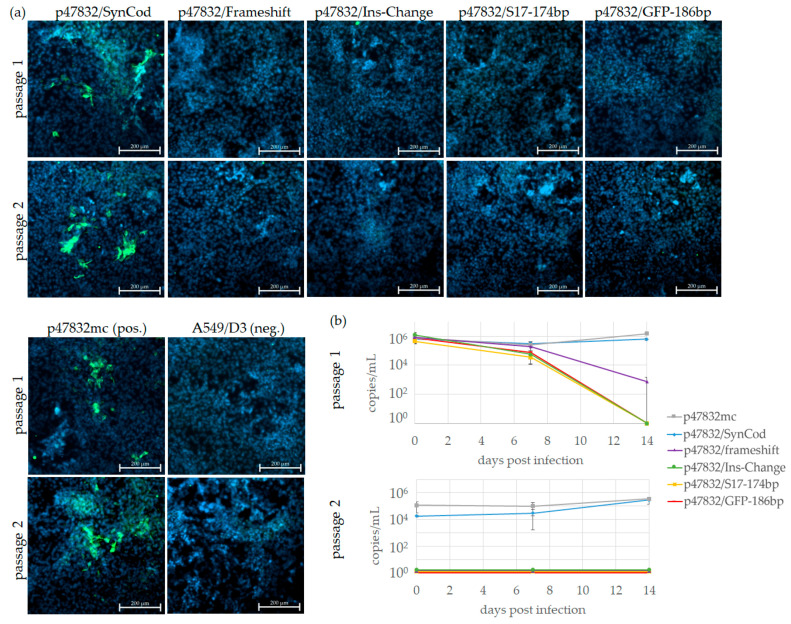
Testing of constructs containing substitutions of the insertion region of the HVR of strain 47832c. The indicated constructs were transfected into BSR T7/5 cells and the resulting cell lysate was used for passaging on A549/D3 cells. The A549/D3 cells from the first (passage 1) and second (passage 2) passage are shown. (**a**) Immunofluorescence analysis of cells using an HEV capsid protein-specific antiserum. Green signal: HEV capsid protein, blue signal: nuclear DAPI staining. Scale bar: 200 µm. (**b**) RT-qPCR analysis of HEV genomes present in cell culture supernatants. Data are means +/− SD from two independent experiments.

Several constructs containing substitutions of the inserted sequence were generated (Figure 1) and tested for their ability to generate infectious virus. The applied method for testing was identical to that described for the deletion mutants. As evident from Figure 3a, fluorescent cells indicating the generation of replicating virus could only be detected in both virus passages for the mutant p47832/SynCod, which has synonymous codons altering the nucleotide sequence, but not the translated amino acid sequence of the insertion. In all other cases (except the positive control p47832mc), no fluorescent cells could be identified in either passage. Additionally performed RT-qPCR analyses of the culture supernatants confirmed this finding, as only in case of p47832/SynCod (and the positive control p47832mc) nearly constant or slightly increasing amounts of the virus genome could be identified in both passages, whereas in the other cases decreasing amounts in the first passage and no genomes in the second passage were detected (Figure 3b). As no rigorous washing steps were performed after inoculation of the wells, residual inoculum may be present in the analyzed samples. In particular, the high amounts of virus-specific RNA at day 0 of passage 1, which were detected for all constructs, presumably originate from transcribed RNA from the transfected plasmids. Therefore, these RT-qPCR data may not accurately reflect the viral growth kinetics, but they can indicate whether there are differences between the individual constructs. The results were confirmed in two independent experiments. This indicates that neither the presence of a frame-shifted (p47832/frameshift) or rearranged RNA sequence (p47832/Ins-change) nor a GFP sequence of the same length as the insertion (p47832/GFP-186bp) or the S17 insertion of strain Kernow C1-p6 (p47832/S17-174bp) was able to generate an efficiently replicating virus using the applied reverse genetics system. In contrast, the presence of other codons, which encode the original amino acid sequence of the insertion, enabled the generation of an infectious virus.

### 3.4. Influence of Specific Glycine-to-Arginine Mutations on Virus Replication

**Figure 4:**
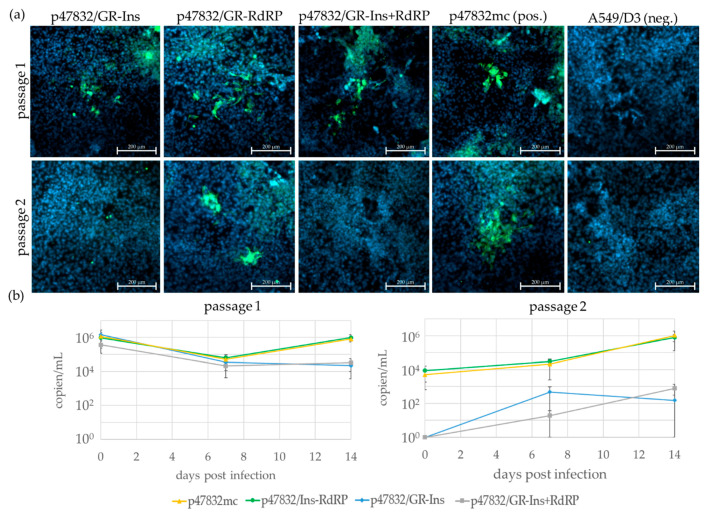
Testing of constructs containing glycine-to-arginine substitutions corresponding to the described G1634R mutation in the RdRp region and/or in the insertion region of the HVR of strain 47832c. The indicated constructs were transfected into BSR T7/5 cells and the resulting cell lysate was used for passaging on A549/D3 cells. The A549/D3 cells from the first (passage 1) and second (passage 2) passage are shown. (**a**) Immunofluorescence analysis of cells using an HEV capsid protein-specific antiserum. Green signal: HEV capsid protein, blue signal: nuclear DAPI staining. Scale bar: 200 µm. (**b**) RT-qPCR analysis of HEV genomes present in cell culture supernatants. Data are means +/− SD from two independent experiments.

The mutation G1634R has been described to enhance the replication of strain Kernow C1-p6 in cell culture [30]. As a duplicate of the corresponding genome region is present in the insertion of the HVR of strain 47832c, the effect of this mutation was analyzed here. To this end, the three mutant constructs p47832/GR-Ins, p47832/GR-RdRp and p47832/GR-Ins+RdRp (Figure 1) were generated and tested for their ability to generate infectious virus using the same method as before. It is evident from Figure 4a that fluorescent cells indicating replicating virus could be detected for all mutants in the first passage. However, in the second passage, fluorescent cells were only detected for the construct p47832/GR-RdRp (and for the positive control p47832mc), but not in the other cases. Analysis of the culture supernatants by RT-qPCR indicated nearly constant or slightly increasing amounts of the virus genome in the case of p47832/GR-RdRp (and p47832c), but slightly decreasing amounts for both other mutants during the first passage (Figure 4b left). In passage 2, the virus genome amount increased for the constructs p47832/GR-RdRp and p47832mc up to 10^6^ genome copies/mL, whereas those of the other mutants only slightly increased up to 10^3^ genome copies/mL (Figure 4b right). Similar to the previous experiment, these RT-qPCR data may not accurately reflect the viral growth kinetics, but they can indicate whether there are differences between the individual constructs. Genome amount differences between day 14 of the first passage and day 0 of the second passage are due to dilution effects, as the latter sample was derived after removal of inoculum and adding of fresh medium (see Section 2.3). These experiments were repeated once with similar results. The results indicate that the introduction of the glycine-to-arginine substitution within the insertion of the HVR of strain 47832c greatly decreases the amount of replicating virus, resulting in an inability for repeated passaging of these viruses in the applied culture system. In contrast, the virus with the glycine-to-arginine substitution in the RdRp region is viable and can be passaged in A549/D3 cells.

### 3.5. Comparison of Growth Kinetics of p47832/GR-RdRp, p47832/SynCod and p47832mc

**Figure 5:**
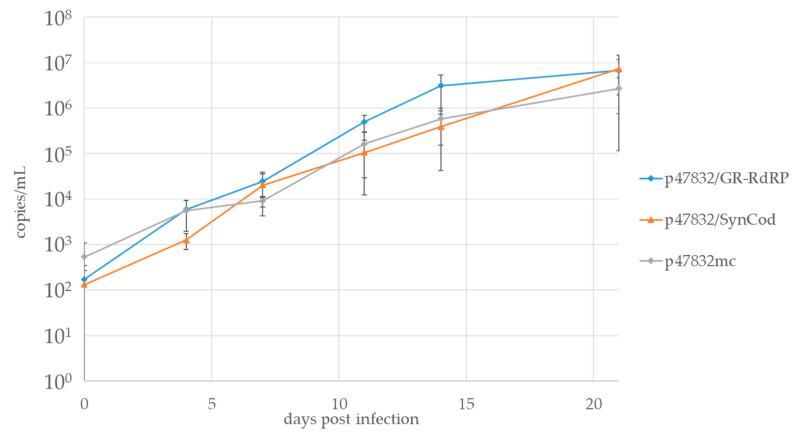
Comparison of viral replication kinetics of the generated infectious virus mutants p47832/SynCod and p47832/GR-RdRp with that of p47832mc. A549/D3 cells were infected with similar virus titers and the culture supernatants were analyzed using RT-qPCR at different time-points after infection. Data are means +/− SD from two independent experiments. An unpaired, heteroscedastic Student’s *t*-test showed no significant differences between virus titers at the different time points (all values *p* > 0.05).

In order to compare the growth kinetics of the generated viruses, which could be passaged twice in A549/D3 cells, culture supernatants containing viruses from constructs p47832/GR-RdRp, p47832/SynCod and p47832mc (see Figure 1) were titrated. Thereafter, fresh A549/D3 cells were inoculated with similar virus titers and the amount of the virus genome in the culture supernatants was monitored using RT-qPCR for up to 21 days after infection. As shown in Figure 5, the virus genome amounts continuously increased from about 10^2^ genome copies/mL to more than 10^6^ genome copies/mL in each case. Although not statistically significant, the virus from the construct p47832/GR-RdRp tended to have slightly higher genome copy amounts than the others at day 11 and day 14 after infection. The presented data originate from two independent experiments.

### 3.6. Testing the Capability of the Constructs to Generate Persistently Virus-Infected Cell Lines

**Figure 6:**
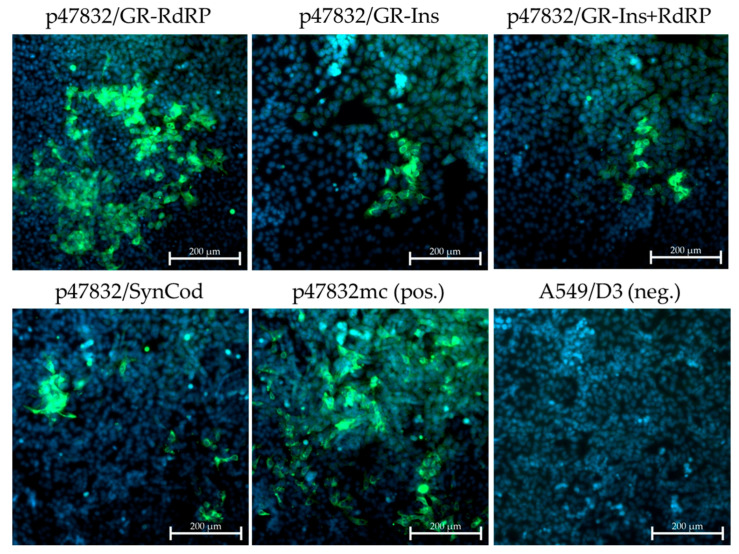
Generation of A549/D3 cell lines persistently infected with viruses generated from the constructs. The indicated constructs were transfected into BSR T7/5 cells and the resulting cell lysate was used for infection of A549/D3 cells. After an initial incubation for two weeks, the cells were split and seeded into new culture flasks, which was repeated every week. Cells from the 4th passage are shown, which were analyzed by immunofluorescence using an HEV capsid protein-specific antiserum. Green signal: HEV capsid protein, blue signal: nuclear DAPI staining. Scale bar: 200 µm.

In the experiments described above, the generated virus had to efficiently replicate within 2 weeks in A549/D3 cells and efficiently released into the supernatant in order to be able to infect fresh cells for the second virus passage. In order to identify slower-replicating viruses, another approach aimed at the development of persistently infected cell lines was therefore tested. To this end, the supernatants from transfected BSR T7/5 cells were used to infect A549/D3 cells similar as in the first approach (with minor modifications due to the use of T25 cell culture flasks instead of 6-well plates). However, after an initial incubation time of two weeks, the cells were split every week in a ratio of 1:2 into new T25 cell culture flasks. After 4 cell culture passages, cells were seeded in 6-well plates and analyzed by immunofluorescence as before. By testing all of the constructs indicated in Figure 1, fluorescent cells were only detected in cells generated with constructs p47832mc (positive control), p47832/SynCod, p47832/GR-Ins, p47832/GR-RdRp and p47832/GR-Ins+RdRp (Figure 6), but not in the other cases. For all successfully replicating viruses, RT-PCRs amplifying the HVR and RdRp region were performed using RNA from the supernatants of the 4th cell culture passage, which were subsequently subjected to Sanger sequencing. By this, the presence of the expected mutations or sequence substitutions was confirmed in all cases (data not shown). The results indicate that in addition to the previously identified replicating viruses from constructs p47832/SynCod and p47832/GR-RdRp, also constructs p47832/GR-Ins and p47832/GR-Ins+RdRp, which harbor the glycine-to arginine mutation within the insertion of the HVR, are capable of slow but stable virus replication using the applied persistent infection approach. A summarizing overview on all results of the experiments performed with all constructs is shown in Table 1.

## 4. Discussion

Insertions within the HVR have been described for several HEV strains detected in chronically infected patients [18,19]. However, the distinct function and mechanism of action of the insertions has not been elucidated thus far, although an involvement in the ability to efficiently replicate in cell culture has been assumed and already demonstrated for some of the strains [21,22]. We investigated the significance of an insertion in strain 47832c for replication in a cell culture system and analyzed the prerequisites of the inserted sequences to support the replication. In contrast to previously investigated strains, the insertion in strain 47832c is derived from different parts of the HEV genome and not from the human host cell genes [21,22,23], which might point to a different mechanism of action.

In a first step, the whole insertion or the two distinct insertion parts were separately deleted in order to analyze their general significance in cell culture replication. As no infectious virus could be generated from these deletion constructs, it has to be concluded that the presence of the complete insertion is essential for virus replication in the used cell culture model. This is in accordance with the findings for strain Kernow C1-p6, which showed markedly reduced virus replication in cell culture after deletion of its inserted sequence [22].

In a second step, the prerequisites of the insertion to support cell culture replication were analyzed using several substitutions of the insertion with other sequences. A substitution of the inserted sequence with an unrelated GFP gene-derived sequence of the same length did not result in the generation of efficiently replicating virus, indicating that the length of the insertion alone is not sufficient for the observed effect. This is also supported by the finding that a construct carrying a changed order of the two parts of the insertion without affecting the overall insertion length did not result in a replicating virus using the applied system. In contrast, this mutant indicates that the specific sequence of the insertion is important for efficient cell culture replication.

In order to analyze if the nucleotide sequence or the translated amino acid sequence of the insertion is important for the observed effect, two complementary constructs were tested. Only the construct retaining the original amino acid sequence by the use of synonymous codons, but not the construct retaining only the original nucleotide sequence by introduction of a frameshift mutation, was able to produce an infectious virus. Of note, this virus with the synonymous codons replicated with similar efficiency to the wild-type virus, indicating that the translated amino acid sequence, not the nucleotide sequence, is responsible for the observed effect. This finding argues against a direct function of the inserted RNA sequence itself, e.g., by providing binding sites for regulatory proteins.

Surprisingly, a substitution of the inserted sequence of strain 47832c with that of strain Kernow C1-p6 did not result in a replicating virus using the applied system. It has to be mentioned that the length of both insertions is somewhat different, which might affect their function in different backbone strains. However, it has recently been shown that the insertion of this S17-derived Kernow C1-p6 sequence into the genotype 1 strain SAR-55 backbone resulted in increased virus replication in hamster cells [22]. Our finding therefore points to a strain-specific activity of the inserted sequence. Alternatively, a cell-type specificity of the insertions could be assumed, as both strains have been analyzed in different cell lines, e.g., the lung carcinoma-derived A549/D3 cells in the case of strain 47832c and the hepatoma-derived HepG2/C3A cells in case of strain Kernow C1-p6 [22]. The growth of the p47832/S17-174bp construct in the hepatoma cells should be investigated in future in order to decide this question. Those investigations should also include testing of complementary constructs containing the insertion of strain 47832c in the backbone of strain Kernow C1-p6 in different cell lines.

The mutation G1634R was originally described in ribavirin-treated patients and was shown to confer enhanced replication in cell culture when present in strain Kernow-C1 p6 [30,37]. Since the respective region is present as a duplication within the HVR insertion of strain 47832c, we initially expected that introduction of this mutation into the inserted region may further enhance virus replication of this strain. However, replicating virus could only be identified in the first virus passage and in the persistent cell line approach, indicating that the mutation in fact greatly decreased virus replication. It is remarkable that a single amino acid substitution in the insertion exerts such a pronounced effect, again confirming the importance of the correct amino acid sequence for its function. As an introduction of the mutation into the original RdRp region of strain 47832c led to a replicating virus, the mechanisms of action therefore seem to be different for the two duplicated regions. The replication kinetics of the 47832c virus with the glycine-to-arginine mutation in the RdRp region were similar or only slightly increased as compared to the wild-type virus. This again points towards a strain- or cell-type-specificity for this mutation, as significantly increased replication enhancement has been described for the Kernow C1-p6 strain with the same mutation [37]. However, it has to be taken into account that the amount of viral RNA was determined in our study, whereas virus infectivity and the amount of the viral capsid protein was assessed in the other study [37], thus making a direct comparison of the data difficult.

Several properties of the insertions in the HVR have been suggested to be responsible for the replication-enhancing effect [18,19]. One observation is that the insertions often lead to a higher charge of the translated protein, because charged amino acids are more often present than non-charged [18,19]. However, the drastic negative effect observed in mutant p47832/Ins-Change, where only the order of the two parts of the insertion were changed, but not the overall amino acid composition, argues against a general role of overall charging. Amino acid sequences, which serve as specific sites for posttranslational modifications or processing of the protein, might also be present in the insertion, the significance of which should be analyzed in future by site-directed mutagenesis. According to amino acid sequence analysis, a sequence potentially functioning as a nuclear localization signal (NLS) can be found in the insertion of strain 47832c, overlapping with the site used for glycine-to-arginine mutation. However, if the NLS is involved in the enhancement of replication, the decreasing effect of this mutation could not be easily explained as the mutation does not change the NLS prediction. Further studies are needed to elucidate the significance of proposed functional motifs of the insertion in future.

## 5. Conclusions

In conclusion, we could show here that the insertion in the HVR of strain 47832c is essential for efficient replication in the cell culture system used in the study. Of note, this insertion is derived from a duplication of the HEV genome and not from human host genes, which have been analyzed in former studies investigating the effects of HVR insertions for HEV cell culture replication [21,22]. By testing several substitution mutants, it was demonstrated here that the translated amino acid sequence of the insertion, but not the RNA sequence, is responsible for this effect. It was also shown that the inserted sequence and a point mutation that led to enhanced replication in strain Kernow C1-p6 did not function similarly in strain 47832c in the tested cell culture system. Therefore, strain- or cell type-specific effects have to be considered. Future investigations should focus on the identification of the distinct mechanism of action of the inserted amino acid sequence for the enhancement of virus replication in cell culture. In addition, the functions of the HVR insertions in chronically infected patients need to be elucidated. These results should help us to understand the basic mechanisms of HEV replication, but could also be used for the generation of faster replicating cell culture-adapted HEV strains, which are urgently needed for basic and applied research on this emerging virus.

## Figures and Tables

**Table 1:** Summary of the generation of HEV 47832c mutants based on the capsid protein detection by immunofluorescence analysis of cells. (+) positive, (-) negative.

Construct *	1st Passage (A549/D3 Cells)	2nd Passage (A549/D3 Cells)	Persistently Infected A549/D3 Cell Line
p47832mc	+	+	+
p47832/Δins 1+2	-	-	-
p47832/ΔIns1	-	-	-
p47832/ΔIns2	-	-	-
p47832/SynCod	+	+	+
p47832/Frameshift	-	-	-
p47832/Ins-Change	-	-	-
p47832/S17-174bp	-	-	-
p47832/GFP-186bp	-	-	-
p47832/GR-Ins	+	-	+
p47832/GR-RdRp	+	+	+
p47832/GR-Ins+RdRp	+	-	+

* The constructs are explained in detail in Figure 1 and Supplementary Table S1.

## Data Availability

Data are contained within the article and supplementary material and available on request from the corresponding author.

## Supplementary Materials

The following are available online at https://www.mdpi.com/article/10.3390/v13050762/s1, Table S1: Details on the generated plasmids carrying deletions, sequence substitutions and point mutations, Table S2: Primers used in this study.
