# Supplementary material for: The Translated Amino Acid Sequence of an Insertion in the Hepatitis E Virus Strain 47832c Genome, But Not the RNA Sequence, Is Essential for Efficient Cell Culture Replication
Source: Viruses. 2021 Apr 26;13(5):762. doi: 10.3390/v13050762 (PMC8145396; doi:10.3390/v13050762)
Supplement: Supplementary file 1 [file viruses-13-00762-s001.zip › viruses-1160397-supplementary.pdf]

viruses

Supplementary Materials: The Translated Amino Acid Sequence of an Insertion in the Hepatitis E Virus Strain 47832c Genome, but Not the RNA Sequence, Is Essential for Efficient Cell Culture Replication

Table S1. Details on the generated plasmids carrying deletions, sequence substitutions and point mutations.

Construct	Nucleotide Position in HEV 47832c (KC618403.1)	Modification	GenBank Accession Number
p47832mc	-	-	MN756606.1
p47832/Δins1+2	2255-2440	Deleted	MW573944
p47832/Δins1	2255-2368	Deleted	MW573945
p47832/Δins2	2369-2440	Deleted	MW573946
p47832/SynCod	2255-2440	Exchanged with synonymous codons	MW573955
p47832/frameshift	2255<br>2440/2441	C deleted<br>C inserted	MW573948
p47832/Ins-Change	2255-2440	Exchanged with nt 2369-2440 followed by 2255-2368 of the HEV 47832c genome	MW573953
p47832/GFP-186bp	2255-2440	Exchanged with GFP-encoding region nt 1166-1351 (MN623123.1)	MW573949
p47832/S17-174bp	2273-2446	Exchanged with human ribosomal S17 subunit (nt 2274-2447 from Kernow-C1, (HQ709170.1))	MW573954
p47832/GR-ins	2375	G exchanged with A	MW573950
p47832/GR-RdRp	5116	G exchanged with A	MW573952
p47832/GR-ins+RdRp	2375, 5116	G exchanged with A, each	MW573951

Table S2. Primers used in this study.

Amplicon	Forward Primer (5′–3′)	Reverse Primer (5′–3′)
Sequencing of HVR fragment	AgCTTATgAAggCTCTgAggTTgA	gTTgggTggAAAgACTCggg
Sequencing of RdRp fragment	gTCCTCTgTAgCgACTACC	AACATgCCAATAAggTTATgTACC
